# Sequential Coupling Shows Minor Effects of Fluid Dynamics on Myocardial Deformation in a Realistic Whole-Heart Model

**DOI:** 10.3389/fcvm.2021.768548

**Published:** 2021-12-23

**Authors:** Jochen Brenneisen, Anna Daub, Tobias Gerach, Ekaterina Kovacheva, Larissa Huetter, Bettina Frohnapfel, Olaf Dössel, Axel Loewe

**Affiliations:** ^1^Institute of Biomedical Engineering, Karlsruhe Institute of Technology, Karlsruhe, Germany; ^2^Institute of Fluid Mechanics, Karlsruhe Institute of Technology, Karlsruhe, Germany

**Keywords:** fluid-structure interaction, multi-physics coupling, cardiovascular modeling, hemodynamics, fluid dynamics simulation, whole heart

## Abstract

**Background:** The human heart is a masterpiece of the highest complexity coordinating multi-physics aspects on a multi-scale range. Thus, modeling the cardiac function *in silico* to reproduce physiological characteristics and diseases remains challenging. Especially the complex simulation of the blood's hemodynamics and its interaction with the myocardial tissue requires a high accuracy of the underlying computational models and solvers. These demanding aspects make whole-heart fully-coupled simulations computationally highly expensive and call for simpler but still accurate models. While the mechanical deformation during the heart cycle drives the blood flow, less is known about the feedback of the blood flow onto the myocardial tissue.

**Methods and Results:** To solve the fluid-structure interaction problem, we suggest a cycle-to-cycle coupling of the structural deformation and the fluid dynamics. In a first step, the displacement of the endocardial wall in the mechanical simulation serves as a unidirectional boundary condition for the fluid simulation. After a complete heart cycle of fluid simulation, a spatially resolved pressure factor (PF) is extracted and returned to the next iteration of the solid mechanical simulation, closing the loop of the iterative coupling procedure. All simulations were performed on an individualized whole heart geometry. The effect of the sequential coupling was assessed by global measures such as the change in deformation and—as an example of diagnostically relevant information—the particle residence time. The mechanical displacement was up to 2 mm after the first iteration. In the second iteration, the deviation was in the sub-millimeter range, implying that already one iteration of the proposed cycle-to-cycle coupling is sufficient to converge to a coupled limit cycle.

**Conclusion:** Cycle-to-cycle coupling between cardiac mechanics and fluid dynamics can be a promising approach to account for fluid-structure interaction with low computational effort. In an individualized healthy whole-heart model, one iteration sufficed to obtain converged and physiologically plausible results.

## 1. Introduction

To be useful in a clinical context, numerical simulation tools always have to strike a balance between high numerical accuracy and low computational effort (1, 2). Especially modeling the human cardiac function *in silico* remains a challenging task: The multi-physics nature of the heart in combination with a multi-scale dimension in time and space claims high demands on computational cardiac modeling (3, 4). As myocardial tension development and wall deformation drive the blood flow, the physical domains of cardiac continuum mechanics and fluid dynamics are of particular interest for the numerical reproduction of the pumping function of the human heart. In this context fluid-structure interaction (FSI) between the heart muscle and the blood flow is particularly important: As the endocardial walls form the physiological boundary layer for the blood, a close interdependence of the concerning quantities is essential for a correct model.

To solve fluid-structure interaction problems two different approaches are common in literature [examples in Hirschhorn et al., (5)]: On the one hand in the monolithic (also called implicit) approach, fluid and solid mechanics equations are computed simultaneously within one solver. Therefore, their mutual influence is directly taken into account, yielding high stability. In that case no explicit coupling algorithm is required as all dependencies are completely modeled in the system of equations. This approach impresses by its high accuracy and inherent inter-dependency of all results. On the other hand the mathematical formulations and their numerical implementation is a challenge. The time step is also restricted due to the requirements of a sufficiently small Courant-Friedrichs-Lewy number (6). Nevertheless, the resulting quantities of both physical domains are immediately available in every time step.

On the other hand partitioned approaches have been suggested (7). Fluid and solid mechanics (the latter in this manuscript referred to as 'mechanics') equations are hereby solved separately, independent of each other by two single solvers. Classically, these two solvers are solved sequentially. Thus, a coupling scheme has to be implemented to account for the mutual influence of the two physical domains. In such a routine information must be communicated between the two solvers in a bi- or mono-directional manner. This approach is computationally more attractive and offers higher flexibility concerning the single solvers. Thus, in each solver the advanced techniques concerning the respective physics can be applied independently of one another (5). However, the required coupling routine has to map data between two temporally and spatially asynchronous grid meshes.

Besides the two basic, purely implicit and explicit algorithms also semi-implicit schemes (8), implicit-explicit variants (9) and multi-way coupled algorithms (10) have been proposed. All in all, a high number of FSI based algorithms and application fields in the cardiovascular system have been published in recent years (5, 11). The number of applications in a clinical context is continuously increasing with computational power and better algorithms. The opportunities and potentials of computational modeling are immense, however not depleted.

Therefore, it is common sense that the fluid-structure interaction takes a central role in reproducing the cardiac function *in silico*. The exchange of information between the single solvers for each different physics is one of the key aspects in the cardiac modeling process. However, the degree to which the spatially resolved blood flow has a retrograde effect on the cardiac mechanics in this multi-physics problem remains unresolved up to now (10). In this study, we focus on the influence of the flow on the local variations of cardiac wall deformation. This is of interest as widely used 3D-0D coupled circulation systems only consider mean spatial chamber pressure values. There, the spatial deviations of the flow velocity field are averaged over the whole chamber. Thus, local increases or decreases are neglected and a unique pressure is considered across the whole chamber. Especially in specific phases of the cardiac cycle like e.g., the ejection phase during the ventricular systole, we hypothesize that this procedure neglects relevant spatial information. In this study, we evaluate this phenomenon quantitatively under physiological conditions.

So in this work we want to find out to which extent the blood flow has a retrograde effect on the structural mechanics. That means we want to answer the question to which extent the force resulting from blood flowing against the myocardial tissue would alter the local deformation of the heart. Following Bernoulli's principle, areas with an increased flow rate are linked to a lower static pressure acting orthogonal to the myocardial wall. Therefore, we expect to find local deviations in the displacement of endocardial wall nodes.

Based on the results of Sacco et al. (12) we assume wall shear stress to be neglectable compared to the internal stresses of the myocardium. Therefore, we analyze the endocardial wall pressure field due to flow dynamics. We subsequently introduce a pressure gradient in the solid mechanical simulation that incorporates the information of the spatially resolved pressure deviations from the mean chamber pressure from the fluid dynamics simulation. This factor is considered in the mechanics simulation to quantify deviations in wall deformation due to the effect of the flow. All in all we want to answer the question whether the influence of fluid dynamics on structural mechanics can be modeled adequately, only by considering information from the pressure field.

## 2. Materials and Methods

### 2.1. Model Generation

All simulations in this study were performed on an individualized whole-heart geometry. The magnetic resonance (MR) imaging data were obtained at Heidelberg University Hospital from a 32-year-old healthy volunteer with a 1.5 T MR tomography system (Philips Medical Systems). The data set was acquired at diastasis and segmented manually. The study was approved by the local IRB, the volunteer gave informed consent.

**Figure 1:**
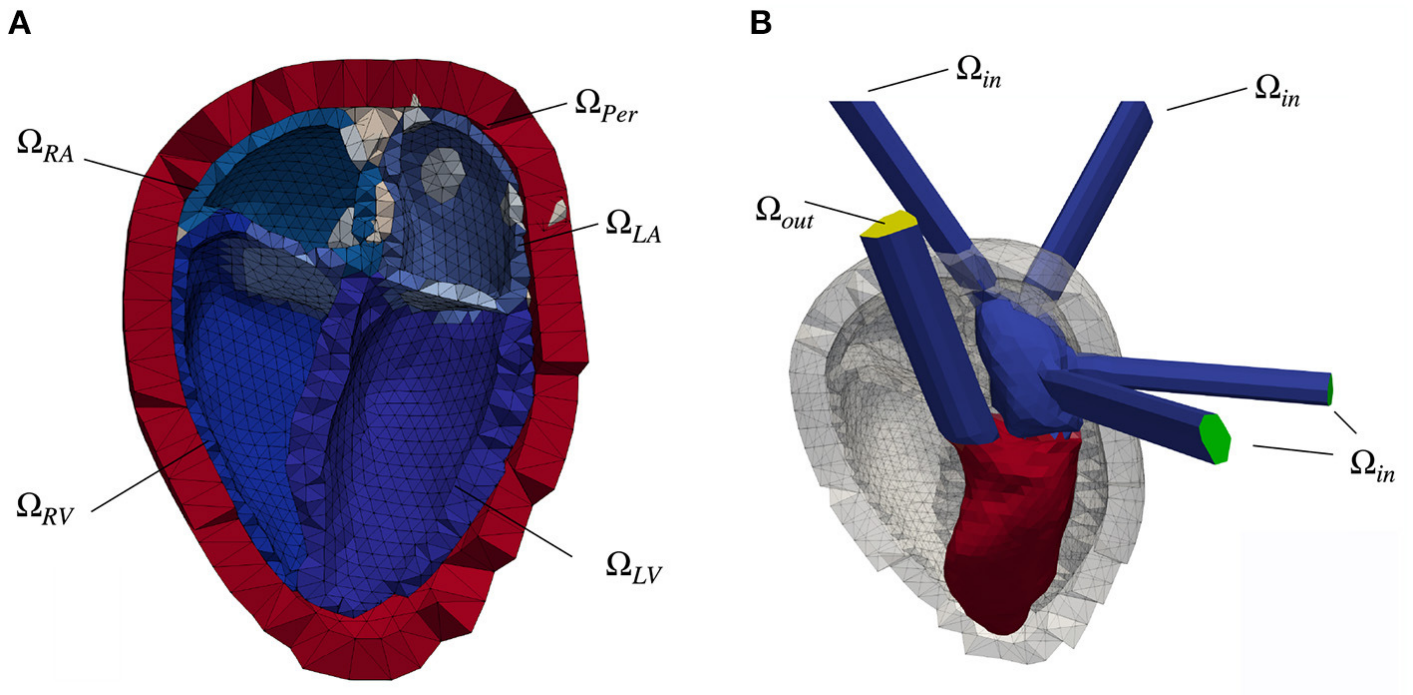
**(A)** Clipped heart geometry with the myocardial tissue of the four chambers colored in shades of blue. The endocardial surfaces of the left atrium (Ω_*LA*_), left ventricle (Ω_*LV*_), right atrium (Ω_*RA*_) and right ventricle (Ω_*RV*_) form the boundary layer for the fluid simulation. Additionally labeled is the pericardial layer (red, Ω_*Per*_) as well as in gray color the area, in which the pulmonary veins (left side of the heart) and the superior vena cava (right heart) open into the corresponding atrium. The blue-gray volume between the atrium and the ventricle denotes the position of the mitral valve (MV, left heart) and the tricuspid valve (right heart). **(B)** Fluid geometry of the left side of the heart with elongated vessel trunks. The pressure inlet surfaces (pulmonary veins) are highlighted in green color, whereas the pressure outlet (aorta) is colored yellow. Also depicted is the initial spatial distribution of the scalar Ψ of the scalar transport equation, color coded in red (Ψ = 1) and blue (Ψ = 0). The gray colored background structures represent the pericardial layer as well as the myocardial tissue in the initial, diastatic state.

To estimate the stress distribution present during image acquisition, a pressure free geometry was estimated by applying an unloading algorithm. Based on the idea of Bols et al. (13), we used the estimation algorithm we presented in Brenneisen et al. (14). Following Peverill (15), a pressure of *p*_0,LV_ = *p*_0,LA_ = 7.5 mmHg was assumed in the left heart chambers during diastatis. For the right heart, we assumed a pressure of *p*_0,*RV*_ = *p*_0,*RA*_ = 4 mmHg. A pericardial layer was used to represent the influence of surrounding tissue (16). A clipped model of the mechanically relevant tissues is shown in Figure 1A.

For the fluid simulations, the pulmonary veins, the pulmonary artery, the aorta and the venae cava were elongated as straight tubes to ensure steady sate flow conditions (Figure 1B).

### 2.2. Structural Mechanics

The mechanical motion of the heart, represented by the spatial displacement of the mesh nodes, is simulated in CardioMechanics (16). The solver was verified in an N-version benchmark study by Land et al. (17). The mechanical finite element mesh comprised 8,700 nodes and 49,900 linear tetrahedral elements. The resulting geometry comprising left ventricle (LV) and left atrium (LA), as well as right atrium (RA) and right ventricle (RV) together with the pericardial layer Ω_*Per*_ is shown in **Figure 1A**.

The myocardial tissue (depicted in different shades of blue in **Figure 1A**) is modeled as a hyper-elastic material like we also did in Kovacheva (18). The passive material properties of the two atria are modeled by the Neo-Hooke material law (19). The ventricular tissue was modeled based on the material law presented by Guccione et al. (20).

The strain energy function *W* for the Guccione material law is given by


(1) $$\begin{array}{ll} W & =\frac{C}{2}\left(e^{Q}-1\right)+\frac{1}{2}K{\left(\det\left(\text{F}\right)-1\right)}^{2} \end{array}$$

(2) $$Q\;=b_{f}E_{11}^{2}+b_{t}(E_{22}^{2}+E_{33}^{2}+E_{23}^{2}$$

(3) $$+E_{32}^{2})+b_{ft}(E_{12}^{2}+E_{21}^{2}+E_{13}^{2}+E_{31}^{2})$$

with the shear modulus *C* and the scaling factors *b*_*f*_, *b*_*t*_, and *b*_*ft*_ being Guccione model parameters. *E*_*ij*_ with *i, j* ∈ [1, 2, 3] denote the elements of the Green strain tensor, det(**F**) the determinant of the deformation tensor. Incompressibility of myocardial tissue was enforced by a penalty formulation (factor *K*) affecting the Jacobian of the deformation gradient. All parameters are chosen as listed in Table 1.

**Table 1:** Passive material parameters used for the different tissue areas in the mechanical simulation.

**Tissue**	**Domain**	**Model**	**C**	***b*_*f*_**	***b*_*t*_**	***b*_*ft*_**	**K**	**ρ_0_**
			**in Pa**				**in kPa**	**in kg m^−2^**
**Ventricle**	LV, RV	Guccione	278	12.0	4.8	8.4	200	1, 082
**Atrium**	LA, RA	Neo-Hooke	7, 450	-	-	-	200	1, 082
**Surrounding**	Pericardium	Neo-Hooke	10, 000	-	-	-	1, 000	1, 082
**Surrounding**	Fat	Neo-Hooke	3, 725	-	-	-	1, 000	1, 082
**Vein**	Pulmonary, vena cava	Neo-Hooke	14, 900	-	-	-	200	1, 082
**Artery**	Pulmonary, aorta	Neo-Hooke	14, 900	-	-	-	200	1, 082
**Valve plane**	Mitral, tricuspid,	Neo-Hooke	200, 000	-	-	-	200	1, 082
	Aortic, pulmonary							

Fiber orientation was determined in a rule-based manner by a Laplace-Dirichlet algorithm suggested by Bayer et al. (21). We used a fiber angle of +60° on the endocardial wall as well as -60° on the epicardial wall.

The active tension development is driven by the double Hill model function introduced by Stergiopulos et al. (22). The periodicity is set to the duration of one heart cycle (*t* = 1.247 s), in accordance with the MRI measurement data.

Taking into account all these influencing factors, the mechanical deformation of the heart geometry is calculated for each point in time. The deformation is the vector that denotes the deviation of each node from the initial state. So this deformation is the effect of the dynamic interplay of active force generated by the contracting cardiomyocytes and the passive material properties that govern elastic deformation.

Coupled to the 3D mechanical solver, a 0D circulatory system was used as presented in Gerach et al. (23) to obtain chamber pressures, flow rates and volumes. The closed-loop, lumped parameter circulatory model accounts for the compliances and resistances of the systemic and pulmonary circulation as well as the behavior of the valves in an electric equivalent circuit. All parameters were applied as presented in Gerach et al. (23). As the four heart chambers are represented by a single variable capacitor in this circuit, the considered chamber pressures are spatial mean values. Thus, for all elements in a chamber, the pressure is the same and no spatial deviations are considered in the mechanical simulation.

The simulation is run for ten full heart cycles in order to reach a limit cycle. In a typical healthy heart cycle, the relaxed state (diastasis) is followed by the atrial systole. With the contraction of the ventricles during the subsequent ventricular systole, blood is ejected through the aortic valve into the systemic circulation. Finally, a relaxation process leads back to the diastatic state and the next cycle follows. The displacement of the endocardial surfaces as well as the pressure of the last heart cycle are extracted as boundary conditions for the fluid solver. In a first step, this serves as a one-way coupled FSI interface as also described in the review by Hirschhorn et al. (5).

### 2.3. Fluid Mechanics

Blood flow was modeled as laminar and incompressible like previously reported in Daub (24). With these assumptions, blood flow velocity *u*_*i*_ and pressure *p* are governed by the Navier-Stokes equations (NSE). To account for the moving mesh, the system of equations is solved in an Arbitrary Lagrangian-Eulerian (ALE) framework, where the mesh motion velocity *c*_*i*_ is included in the divergence term of the NSE (25). This yields the equation


(4) $$\frac{\partial u_{i}}{\partial t}+u_{j}\frac{\partial (u_{i}-c_{i})}{\partial x_{j}}=-\frac{1}{\rho }\frac{\partial p}{\partial x_{i}}+\frac{\mu }{\rho }(\frac{\partial^{2}u_{i}}{\partial x_{j}\partial x_{j}}),$$

in which blood material parameters are chosen equal to those previously reported in Daub (24) (density ρ = 1, 055kgm^−3^ and dynamic viscosity μ = 0.004kgm^−1^s^−1^). Index *i* denotes the respective equation for the concerning coordinate direction. During the solution procedure, the spatially resolved pressure is obtained for each element using a PIMPLE loop [PISO algorithm (26) with iterative marching].

The fluid solver is based on a finite volume representation of the mesh accounting for the blood volume. The left heart as well as its embedding in the surrounding layers (gray color) is shown in Figure 1B. At the endocardial boundary, the wall movement is prescribed based on the solution of the structural mechanics simulation in the current iteration. The displacement of the nodes on the endocardium is provided as a time series for one full heart cycle. A Laplace equation with quadratic diffusivity is solved to determine the mesh motion velocity of the inner volume cells. Hence, a no-slip condition is applied at the ventricular wall for both velocities: the blood velocity, *u*_*i*_, and the mesh motion velocity, *c*_*i*_.

The flux boundary conditions at the inlets Ω_*in*_ and outlets Ω_*out*_ are Neumann boundary conditions, such that the global volume flux, $\overset{∙}{V}$, is in accordance with the volume change prescribed by the boundary conditions from the mechanics simulation. The inlet pressure is prescribed at the pulmonary veins. The outlet pressure is prescribed at the aorta. These Dirichlet type boundary conditions are provided by the circulatory system model.

The tetrahedral fluid volume mesh consist of 143, 000 cells in the left heart, which corresponds to the number of cells found to be sufficient by Schenkel et al. (27). The simulations start with an initial time step of Δ*t*_0_ = 0.001 s. Afterwards, the time stepping is adjusted automatically to satisfy a Courant-Friedrichs-Lewy number of *CFL* ≤ 0.7. At the initialization of the solver, the fluid is at rest. Four consecutive heart beats are simulated to converge to a limit cycle (periodicity of the flow). Finally, the last cycle is used for the coupling with the structural mechanics solver.

All four valves are modeled by means of a porous material law that generates a pressure drop across the respective valve plane as shown in Daub et al. (28). Since the whole heart and therefore also the valve planes are moving with time, the porosity model developed by Wang et al. (29) is incorporated. It considers an increased kinetic energy term in the NSE based on the mesh displacement. Hereby, the porosity model forces the velocity within the porous zone to be equal to the motion of the respective plane:


(5) $$\begin{array}{l} F_{p}=-\frac{\mu }{\rho }\frac{\phi_{p}}{k_{p}}\cdot \left(u_{i}-c_{i}\right)-\frac{1.75\sqrt{\phi_{p}}}{\sqrt{150k_{p}}}\cdot \left(u_{i}-c_{i}\right)|u_{i}-c_{i}|. \end{array}$$

**Figure 2:**
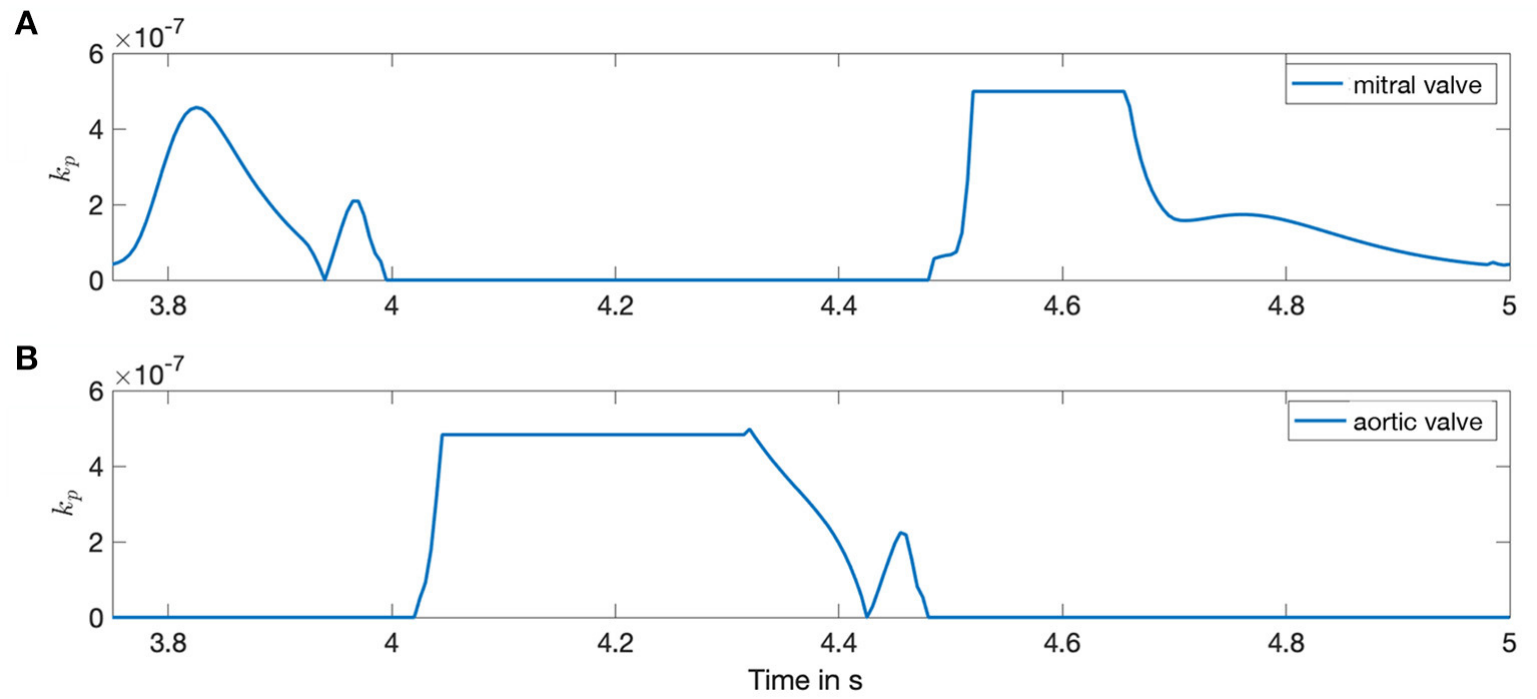
Porosity of the valves during the time course of one heart cycle. Both sub-figures show the valve plane permeability *k*_*p*_ for the left side of the heart. Horizontal lines depict a fully opened or closed valve state. **(A)** Mitral valve. **(B)** Aortic valve.

The resultant forcing term *F*_*p*_ is inserted into the NSE (Equation 4) and adjusts each time step dependent on *c*_*i*_. The valve plane permeability *k*_*p*_ varies between infinitesimally small (impermeable) and 1 (permeable). The respective condition is changed dependent on the ventricular volume change resulting from the boundary conditions such that all valve planes are blocked if $|\overset{∙}{V}|<20mls^{-1}$. Up to a flux of $|\overset{∙}{V}|<160mls^{-1}$, the permeability rises linear with the flow and the valve plane is fully permeable if $|\overset{∙}{V}|>160mls^{-1}$. The same limits in reverse order apply for the closure of the valves. The porosity ϕ_*p*_ = 1 is kept constant. Exemplary, the valve plane permeability *k*_*p*_ is shown in Figure 2 for one heart cycle.

Based on the mitral valve diameter *d*_*MV*_ = 15 mm, the Reynolds number reached at maximum velocity *u*_*max*_ in the valve plane is *re*_*max*_ = *U*_*max*_ρ*d*_*MV*_/μ = 2, 760. The Womersley number lies in the physiological range (30), as $wo=d_{MV}\sqrt{\omega \rho /\mu }$ = 19.3, where ω represents the angular frequency.

All simulations are executed in the open-source software framework openFOAM under the version v1912 (31). We simulated a series of four full heart cycles to reach a limit cycle.

### 2.4. Fluid-Structure Coupling

**Figure 3:**
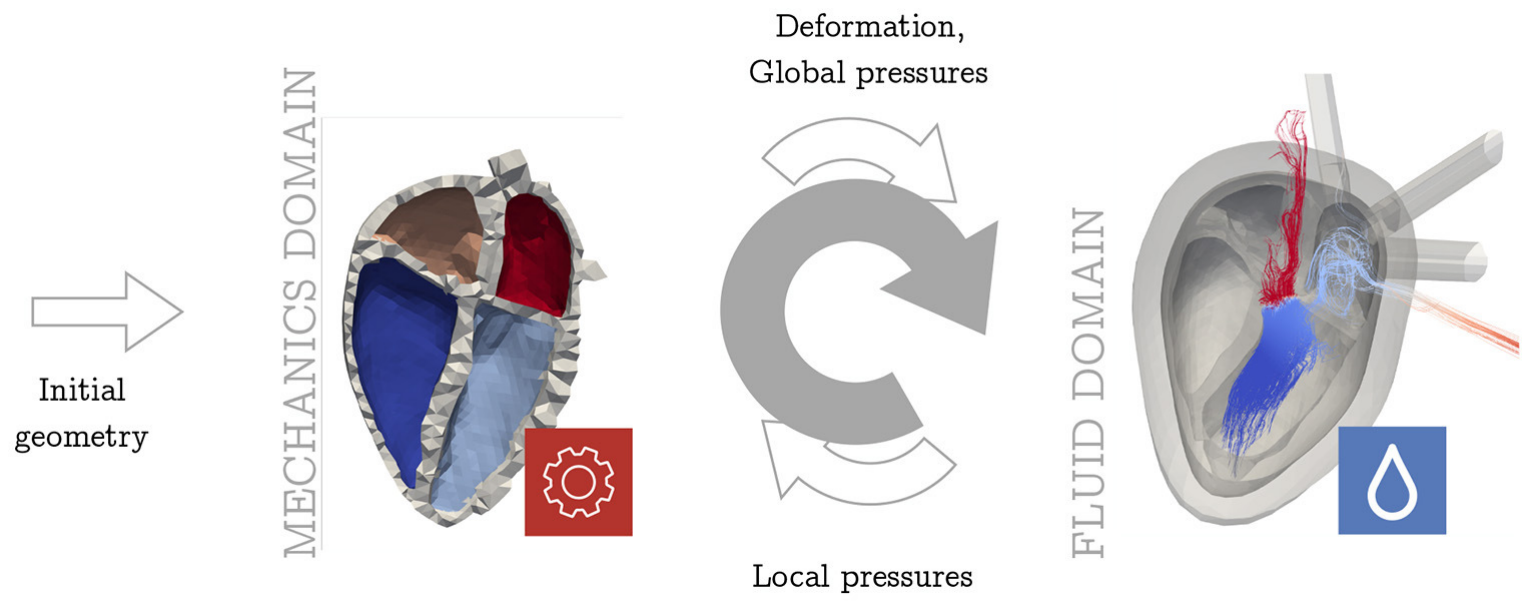
Overview of coupling procedure. The arrows denote the flow of information between the two domains. The upper arrow shows the unidirectional information flow from the mechanical domain to the fluid solver as it was implemented before [e.g., Daub et al. (28)]. Now the lower arrow takes into account the retrograde effect from fluid dynamics in the mechanical simulation. This sums up to an iterative sequential procedure (gray arrow). Both—in the mechanics and in the fluid domain—several full heart beats are simulated before handing over the data to the other domain.

As discussed in the introduction, different approaches for FSI have been suggested in literature. To account for the influence of FSI in a computationally and structurally beneficial way, we propose a cycle-to-cycle coupling as visualized in Figure 3 to quantify the effects of fluid dynamics on the mechanical results. In this section, we first introduce the pressure factor *x* that is used to communicate the spatially resolved information from the fluid simulation back to the solid mechanical simulation. Secondly, we present the complete overview over the whole coupling procedure.

Toward this end, we extract the pressure *p*_*i*_ with *i* ∈ *N* for all *N* endocardial wall elements from the three-dimensional pressure field *p*. As reported in Brenneisen et al. (32), we introduce a 3D spatially resolved pressure factor *x*. Compared to the previously published version, we introduce an additional scaling factor *y*_*s*_ to weigh the influence of the relative pressure factor based on the absolute pressure values *p* in the fluid mechanics simulation:


(6) $$\begin{array}{l} x_{i,t}=y_{s,t}\cdot \frac{p_{i,t}-p_{mean,t}}{p_{max,t}-p_{min,t}}+1 \end{array}$$

for each element *i* at the time *t*. The statistical values minimum (*p*_min_), maximum (*p*_max_) and mean (*p*_mean_) pressure are evaluated spatially for the concerning heart chamber at the current time *t*.

The subtraction of the mean pressure *p*_mean_ and the choice of the denominator in equation 6 ensures that the mean value of the pressure factor is equal to one. Therefore, the equation


(7) $$\begin{array}{l} \frac{1}{N_{c}}\overset{N_{c}}{\underset{i=1}{\sum }}x_{i,t}=1 \end{array}$$

is fulfilled for each of the four heart chambers, where *N*_*c*_ is the number of surface elements in the corresponding heart chamber *c* with *c* ∈ {LA, LV, RA, RV}.

This definition of a mean-free pressure factor is consistent with the circulatory system model, which delivers one average pressure *p*_*mech,c*_ for each heart chamber. Thus, the pressure conditions in the circulatory system model do not get out of balance due to the influence of the fluid solver.

For a scaling factor *y*_*s*_ = 1, the dimensionless pressure factor *x* ranges between zero and two with numbers between one and two for a fluid pressure higher then the mean pressure provided by the circulatory system.

However, a static scaling factor *y*_*s*_ = *const*, that maps all element pressures *p*_*i*_ on the scaling factor interval *y*_*s*_ ∈ (0 … 2) is not capable to fully reproduce the absolute pressure ranges.

Therefore, we introduced the time-resolved scaling factor


(8) $$\begin{array}{l} y_{s,t}=2\cdot \frac{p_{max,t}-p_{mean,t}}{p_{max,t}} \end{array}$$

also based on the statistic quantities of the fluid simulation. This choice ensures that a multiplication of the scaling factor *x* with the mean pressure *p*_*mean*_ reproduces the fluid pressure *p*_*i*_ without changing the mean pressure factor *x*_*mean*_ = 1. In order to ensure the robustness of the procedure, the scaling factor is limited to the range *y*_*s*_ ∈ (−3, 3).

Finally, the computed pressure factor *x* serves as an input to the subsequent mechanical simulation, thus closing the loop of the iterative coupling procedure.

The adapted mechanical pressure $p_{mech}^{*}$ is calculated by a multiplication of the pressure estimated by the circulatory system with the pressure factor *x* following equation


(9) $$\begin{array}{l} p_{mech,i,t}^{*}=p_{mech,c,t}\cdot x_{i,t}. \end{array}$$

**Figure 4:**
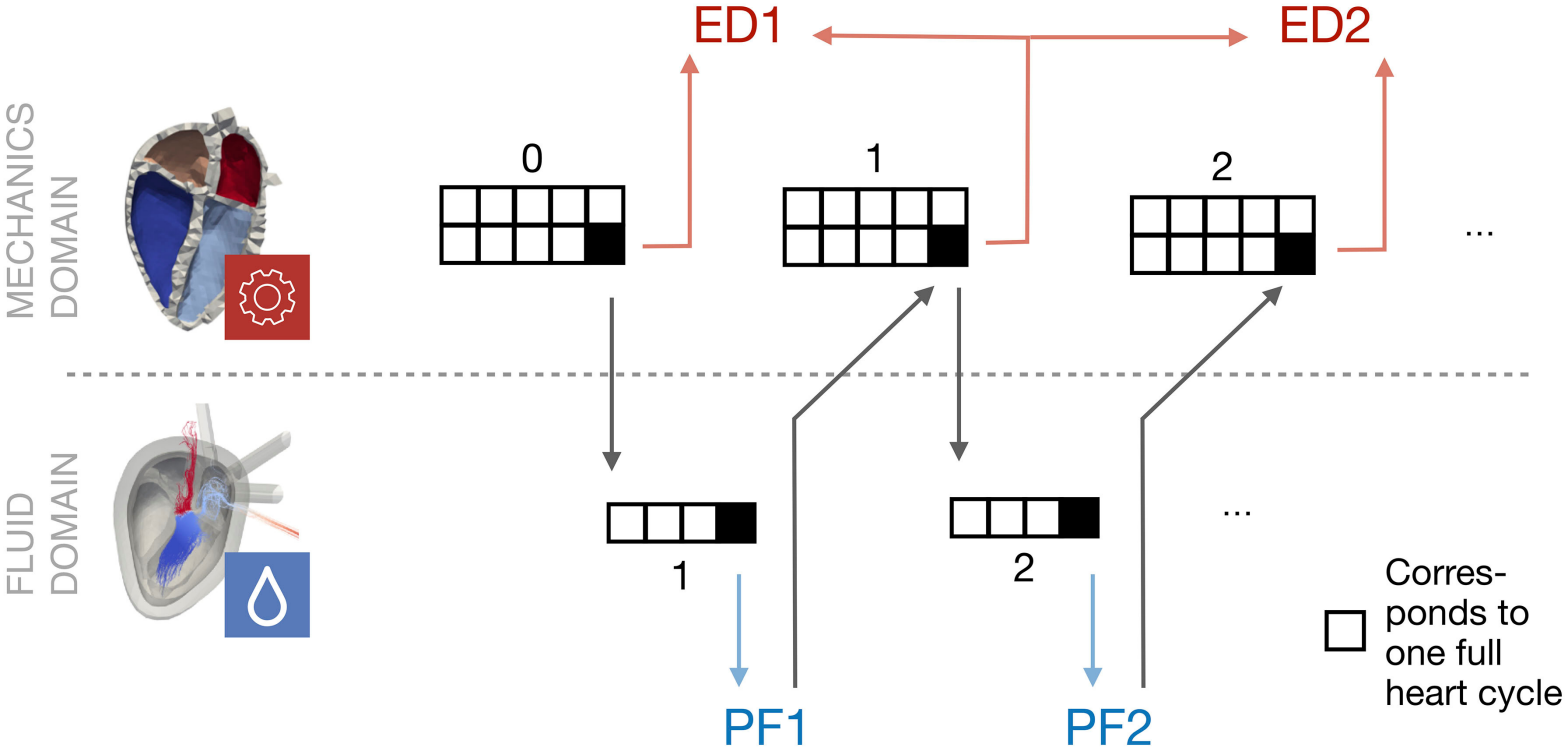
Schematic overview of the coupling procedure. Starting with a mechanical simulation, the last heart cycle deformation is used as a boundary condition for the subsequent fluid simulation. The processed pressure factor (PF) is used as an input in the next mechanical simulation, when it has reached a steady state. This procedure is repeated. The Euclidean distance (ED) is evaluated between the last cycle of two subsequent mechanical simulations.

Figure 4 shows a schematic overview of the coupling procedure. The initial mechanical simulation is run for ten heart cycles to reach a steady state. The deformation, i.e., the coordinates of the endocardial surface of the last heart cycle is extracted. Also the pressure at the pulmonary veins and the aorta is extracted from the circulatory system for the last heart cycle. These quantities are respected as a boundary condition in the fluid simulation. After four cycles of fluid simulation, a limit cycle is reached. The spatially resolved pressure field is extracted. For all elements on the endocardial wall, the pressure factor *x* is calculated following equation 6. This pressure factor is then respected as a boundary condition in the last cycle of the subsequent solid mechanical simulation. The number of ten mechanics simulations is also applied in this stage to ensure that all transients have decayed. This procedure is applied iteratively.

For this study, *N*_*L*_ ≈ 2, 200 endocardial surface elements were evaluated in the left heart (*N*_*LV*_ ≈ 1, 500 and *N*_*LA*_ ≈ 700).

### 2.5. Evaluation Criteria

To evaluate the convergence of the proposed coupling algorithm, three criteria are investigated. First, the mechanical deformation difference between the different iterations is analyzed. Additionally, a scalar transport equation to evaluate fluid simulations is introduced. Finally, simulation results are evaluated based on pressure-volume loops.

#### 2.5.1. Deformation

To evaluate the effect on the mechanical simulation, the displacement of the endocardial wall throughout a heart cycle is analyzed. Therefore, the Euclidean distance (ED) *d* is calculated:


(10) $$\begin{array}{l} d_{i,j}=\sqrt{{\left(x_{i,j+1}-x_{i,j}\right)}^{2}+{\left(y_{i,j+1}-y_{i,j}\right)}^{2}+{\left(z_{i,j+1}-z_{i,j}\right)}^{2}} \end{array}$$

for each node *i* in time and space, based on the node location (*x, y, z*) in the two subsequent iterations *j* and *j* + 1.

#### 2.5.2. Hemodynamics

As a measure for the fluid simulation results with diagnostic value, the mixing procedure of blood in the ventricles is analyzed by solving a scalar transport equation. It is a convection-diffusion equation suggested by Ferziger et al. (33) and it accounts for the passive transport of the scalar Ψ by the velocity field *u*. Ψ can therefore be considered as the local concentration of initial blood and enables a tracking of residual blood volume. The passive transport equation


(11) $$\frac{\partial \Psi (x,t)}{\partial t}+U_{i}(x,t)\frac{\partial \Psi (x,t)}{\partial x_{i}}-\frac{\partial }{\partial x_{i}}(D\frac{\partial \Psi (x,t)}{\partial x_{i}})=0$$

with *i* ∈ {1, 2, 3} also comprises an artificial diffusion coefficient *D* = 10^−10^m^2^s^−1^ for numerical stability. For *t*_0_ = 0 s, the scalar Ψ is initialized with


(12) $$\Psi (x,t_{0})=\{\begin{array}{ll} 1 & \text{for all elements in the LV }\\ 0 & \text{elsewhere} \end{array}$$

The distribution of Ψ in the initial state is shown in Figure 1B.

## 3. Results

Following this sequential coupling approach, we simulated three iterations of mechanics simulations and the associated two fluid simulations like we showed in Figure 4. In this section we present the results of the introduced pressure factor as well as the Euclidean distance as the performance measure. Finally, the influences onto the fluid domain as well as the pressure-volume loops are presented.

### 3.1. Pressure Factor

**Figure 5:**
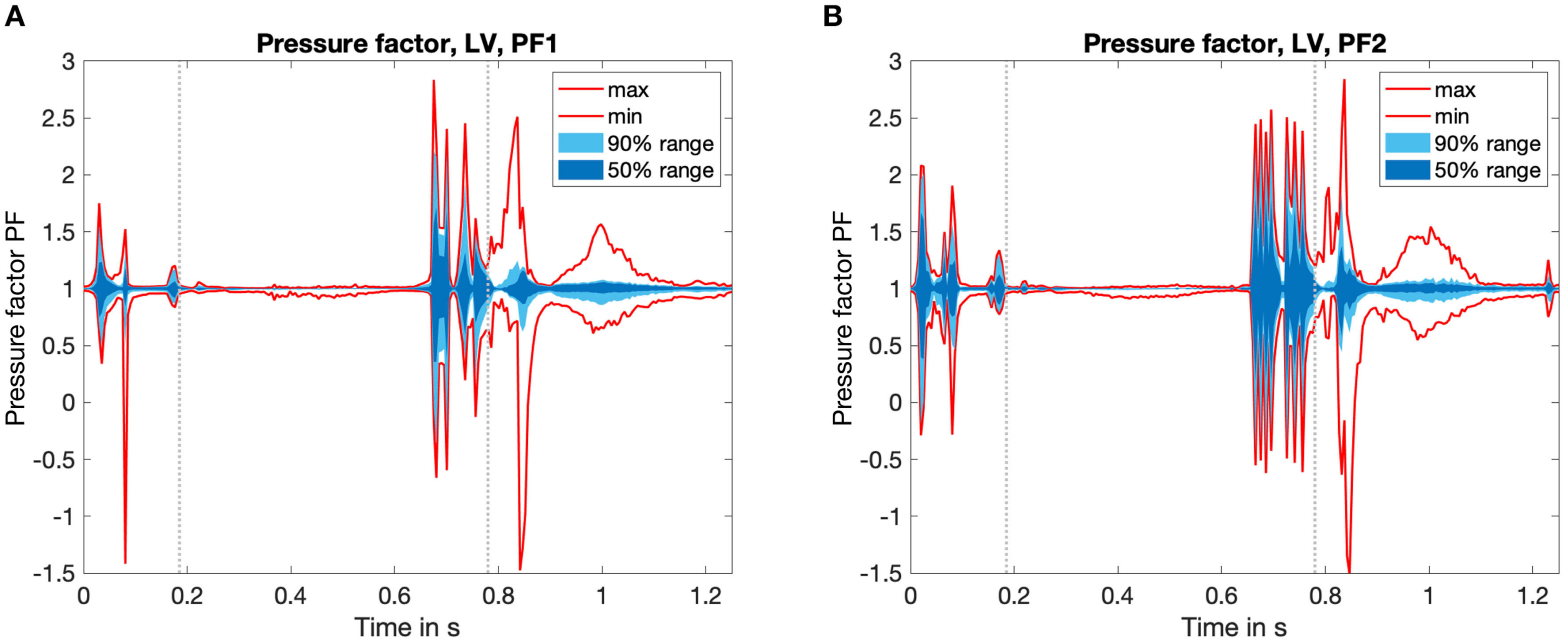
Time course of the pressure factor PF over one heart cycle. Calculated by Equation 6, it visualizes the influence of the fluid dynamics simulation. Values *PF* > 1 denote a fluid pressure higher than the one calculated by the circulatory system. The statistical distribution of the minimum and the maximum value, as well as the area which covers 50 and 90% of all values are highlighted in red as well as dark and light blue color. For temporal orientation, the vertical gray lines mark the start and the end of the systolic phase. **(A)** First iteration of the fluid solver. **(B)** Second iteration of the fluid solver.

To investigate the pressure deviations in the fluid simulation, we evaluated the time course of the normalized pressure factor in Figure 5. The first fluid simulation is shown in Figure 5A, the second iteration in Figure 5B. The pressure factor is in the range of −1.5 < *PF* < 3 for both simulations. The mean value is *PF*_*mean*_ = 1 per definition, but also the 50% quantile is pretty close to *PF*_*mean*_ = 1 throughout most of the time. At two characteristic points of the heart cycle, higher deviations can clearly be observed: On the one hand, at the beginning of the heart cycle (*t* = 0.1 s) and on the other hand during the ventricular systole (*t* = 0.7 s). The highest deviations occur during systole close to the aortic valve.

**Figure 6:**
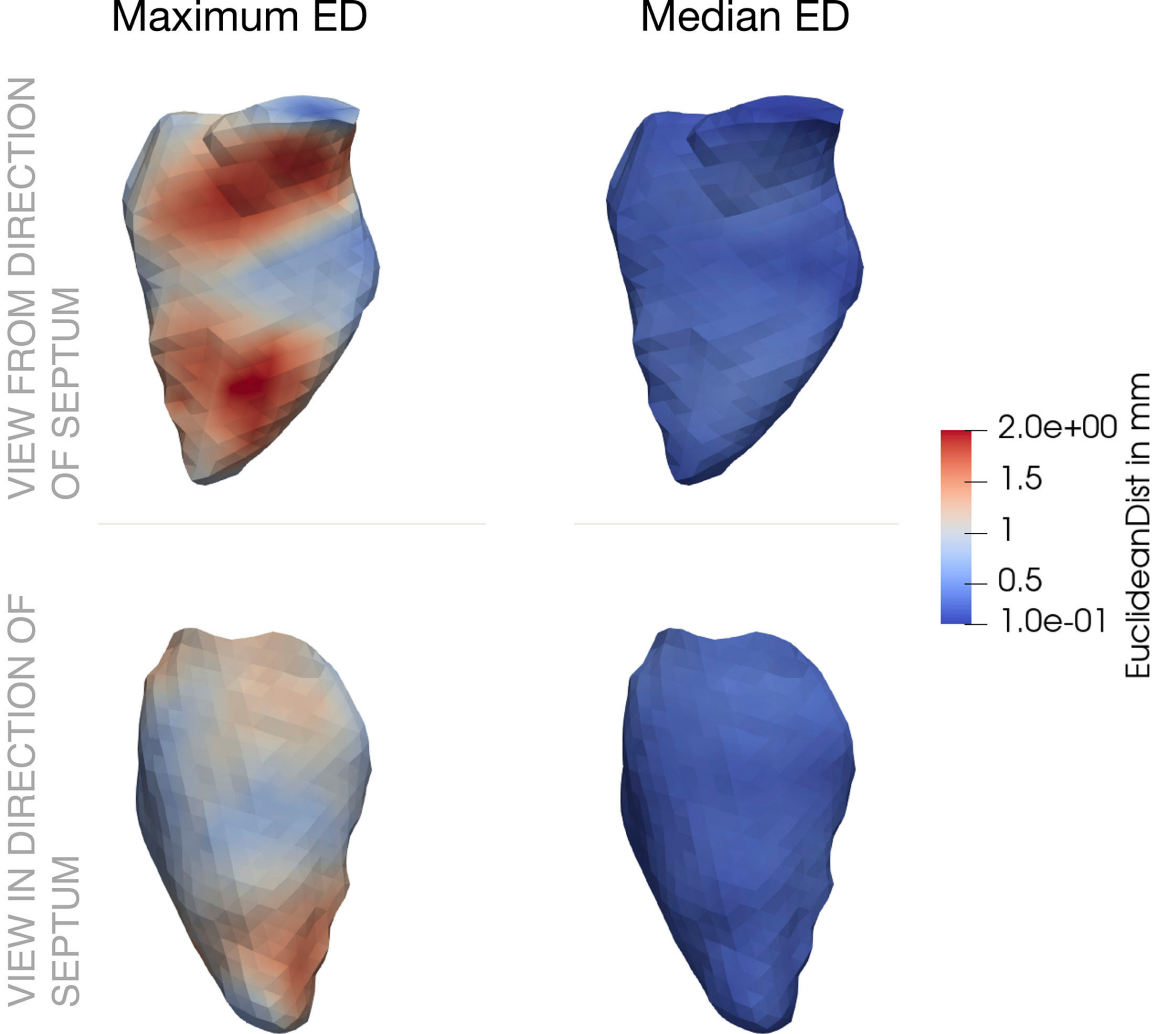
Spatial distribution of Euclidean distance (ED) across the left ventricle. The first column shows the maximum values, the second column the median values. In the upper row, the ventricle is visualized from a septal position, the bottom row shoes the opposite view. ED between the initial mechanical simulation (*j* = 0) and the subsequential one.

If we investigate a spatially resolved map of the pressure factor, it is clearly visible that the large pressure deviations occur close to the aortic valve, where blood is ejected during systole. Thus, a pressure higher than the mean pressure of the chamber (which is the one corresponding to the boundary conditions from the mechanical simulation) is present. This is also accompanied by the spatial distribution of the Euclidean distance in Figure 6.

While comparing the two fluid iterations shown in the overview Figure 4, we found that the basic shape of the PF time course stayed equal throughout the plots of Figures 5A,B. On the one hand, this confirms a consistent behavior of the coupling algorithm: The timing of the coupling framework matches the two single physics solvers. On the other hand the reduced amplitude of the pressure factor by around 10% shows the tendency to converge toward a limit cycle.

### 3.2. Deformation

**Figure 7:**
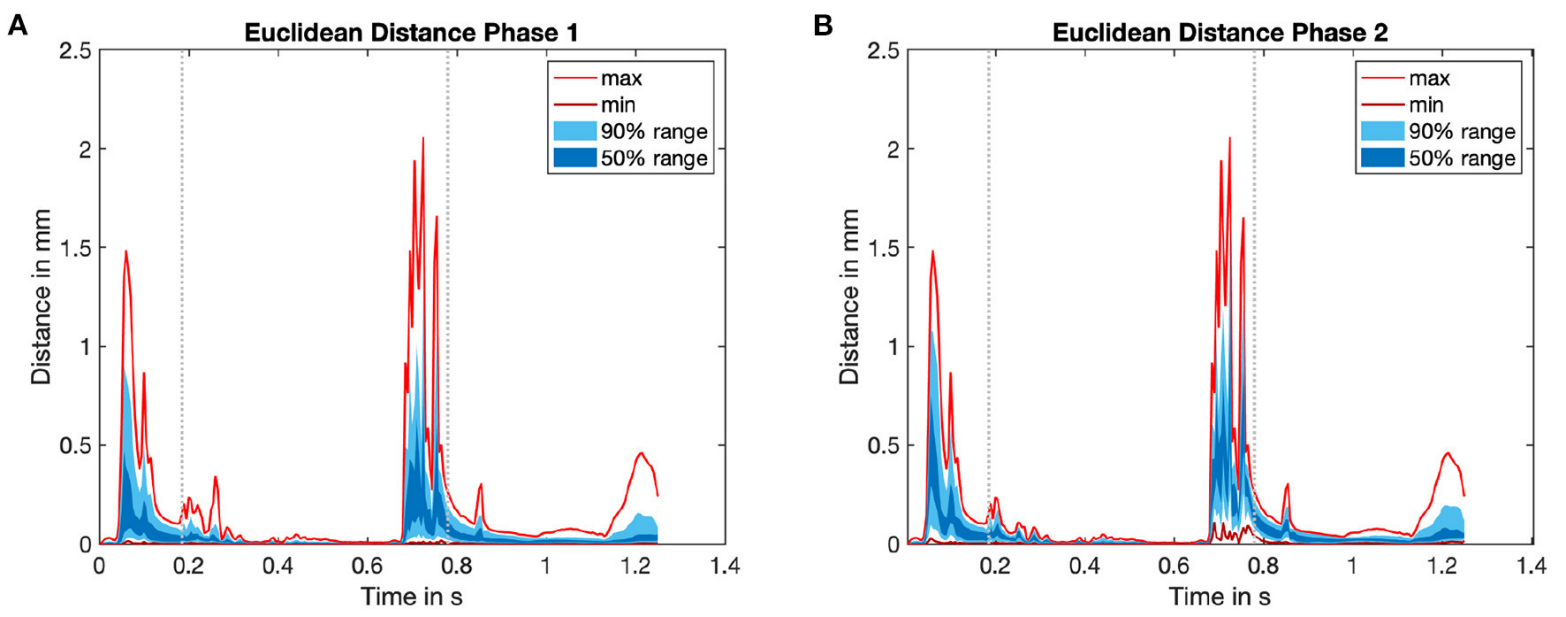
Time course of Euclidean distance (ED) for the last heart cycle in every iteration of the mechanical solver. For an overview see again Figure 4. The statistical distribution of the minimum and the maximum value, as well as the area which covers 50 and 90% of all values are highlighted in red as well as dark and light blue color. **(A)** ED0: ED between initial mechanical simulation (*j* = 0) and subsequent iteration (mechanical simulation 1). **(B)** ED1: ED between mechanical simulation 1 (*j* = 1) and subsequent mechanical iteration. See Figure 4 for an overview.

In the second step we evaluated the Euclidean distance (ED) between the corresponding nodes of the mechanics simulation in subsequent iterations. The time course of the overall ED for the last heart cycle is shown in Figure 7. For the left ventricle (LV), the maximal ED of all endocardial wall nodes was smaller than 2 mm between the first and second iteration. The maximal distance between the second and third iteration was 70 μm, thus the limit of necessary cycles was already reached after two iterations.

The spatial distribution of the ED throughout the LV is shown in Figure 6. In the left part, the maximum ED is shown from the direction of the septum (upper part), as well as from the opposite side (lower part). The maximum deviation clearly occurs in the area of the aortic valve and close to the apex. These two locations correspond to the two characteristic points in the cardiac cycle: While blood flows into the ventricle during atrial contraction, the jet toward the apex increases the pressure. On the other hand, during ventricular systole, blood moves toward the aorta and increases the pressure in this area.

Comparing to the right part of Figure 6 reveals that the median ED is distributed uniformly across the LV and is in the range below 1 mm. Minimum, maximum and mean absolute numbers for the ED are listed for two iterations in Table 2.

**Table 2:** Euclidean distance in mm.

**Euclidean distance ED**	**Left ventricle (LV)**			**Left atrium (LA)**			**Overall**		
**in mm**	**min**	**max**	**mean**	**min**	**max**	**mean**	**min**	**max**	**mean**
Iteration 0/1 (j=0 / ED0)	0.00	2.07	0.06	0.00	2.05	0.03	0.00	2.07	0.04
Iteration 1/2 (j=1 / ED1)	0.00	2.06	0.06	0.00	1.66	0.03	0.00	2.06	0.04

*It is calculated as the deviation of a node comparing two subsequent iterations of the mechanical solver. In this case, the ED between iteration 0 and 1 (j = 0) as well as the ED between iteration 1 and 2 (j = 1) are given and quantified for the three regions LV, RV and the overall geometry. Minimum, mean and maximum values are listed*.

These results show that the changes in deformation as a reaction to the pressure gradient distribution are small but still exist.

Comparing the right part of the time course in Figure 7 reveals that a limit cycle is already reached after one iteration of the fluid solver.

### 3.3. Hemodynamics

**Figure 8:**
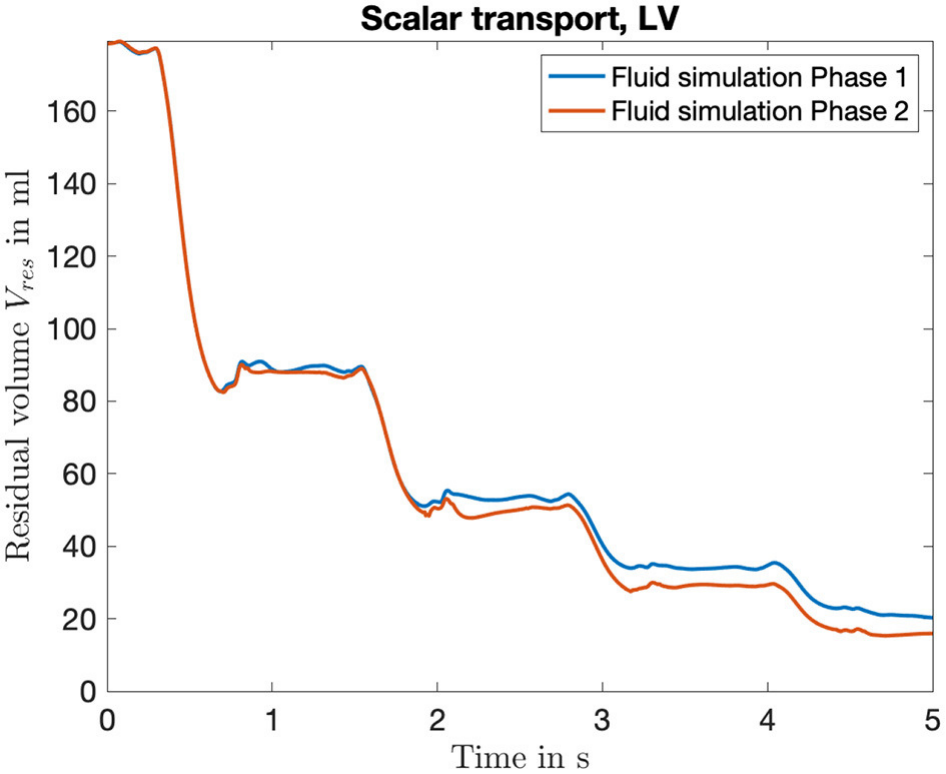
Time course of the residual volume introduced to quantify the washout during the fluid simulation. It is computed based on the transport of the scalar Ψ, which can be seen as a local concentration of initial blood volume in the LV. Shown is the curve for the four full heart cycles, color coded are the two iterations of the fluid solver.

To evaluate the effects of the coupling on the fluid dynamic quantities, we investigated the residual volume *V*_*res*_ in the left ventricle. The time course of four heart cycles is shown in Figure 8. Hereby, the residual volume is calculated by a multiplication of the scalar Ψ computed by the passive scalar transport (equation 11). Therefore, the blood volume that initially (at *t* = 0 s) started in the LV and still remains there is depicted in milliliter. If the influence of the pressure factor is not yet considered (Fluid simulation Phase 1), a residual volume *V*_*res*,1_ ≈ 20 ml is achieved. Considering the influence of the pressure factor, a residual volume of *V*_*res*,2_ ≈ 16 ml results. So all in all a difference of Δ*V*_*res*_ = 4 ml, compared with a *V*_*ED*_ ≈ 180 ml describes a deviation of approximately 2%. Therefore, the slight changes in deformation of the boundary surface reveal a small but not neglectable accumulated residual volume.

### 3.4. Pressure-Volume Loop

**Figure 9:**
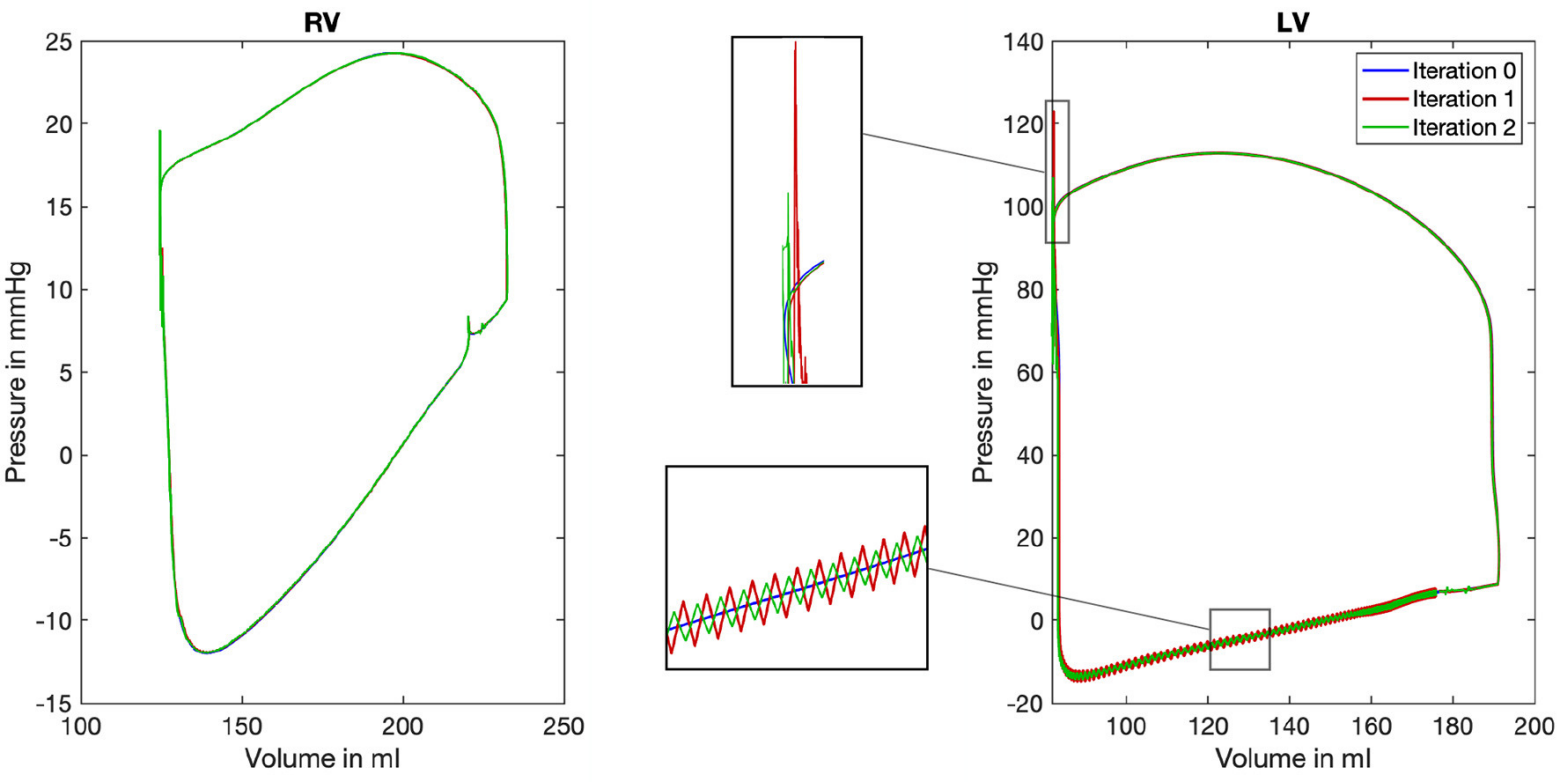
Pressure-volume loop for the right (RV) and left (LV) ventricle. Color coded are the three iterations of the mechanical solver while the first iteration depicts the limit cycle after nine iterations of the mechanical solver (mechanical simulation 0, blue color). For the LV, two characteristic areas are shown enlarged in the center of the figure to highlight the differences between the three iterations.

Pressure-volume (PV) loops are often used to represent the work and efficiency of a mechanical system. The same holds true for the human heart: The PV-loops in Figure 9 show the mean ventricular chamber pressure plotted against the ventricular chamber volume and reveal the influence of our coupling framework. As the mean pressure of each chamber *p*_*mean,c*_ is per definition kept constant (equation 7), deviations in the PV-loop are only caused by an altered chamber volume. Therefore, the changes in the diagram are not that great. However, some deviations exist and a low alteration of the chamber volume can be found. While the blue line shows the resulting PV-loop after the nine heart cycles of mechanical simulations (limit cycle, compare overview in Figure 4), the red and green lines show the result of considering the pressure factor. Deviations can clearly be observed in the lower left part of the curve. At this point in time, the isovolumetric relaxation (left vertical line) and the diastolic filling (lower horizontal line) take place.

Comparing the PV-loops of the two iterations again confirms the convergence of the system toward a limit cycle: Deviations in the chamber volume become smaller. Furthermore, it can be recognized that the deviations in the mean chamber volume are below Δ*V*_*c*_ < 1 ml at every point in time.

However, while comparing the initial iteration (blue) to the first (red) and second (green) also reveals differences: On the one hand, at the end of the systole, a pressure peak can be observed in the top left area of the loop. On the other hand, especially in the lower and left part of the loop, oscillations are introduced by the iterative coupling procedure. However, we observe that the amplitudes of both artifacts decrease in the second iteration.

## 4. Discussion

The novel coupling approach combines realistic feedback with comparably low computational effort and independence of the two single physics solvers. The coupling interface can be used independently from the exact solver architecture and therefore offers a high degree of flexibility and applicability. It unites simulation software packages focusing on only one physics to exchange relevant information in a bidirectional manner. In this way, we enable to consider fluid dynamic influences in structural mechanics simulations without a need of direct interaction during the simulation itself.

The main task and also the biggest challenge that partitioned FSI solvers face is the adequate exchange of coupling data. In most cases, different underlying mesh geometries have to be compared and resulting quantities have to be mapped and interpolated onto the concerning meshes. This process highly relies on the accuracy of all models, as well as a correct mapping. Therefore, coupling frameworks are prone to introduce errors (5). Since in our approach, the solvers do not exchange information in each time step Δ*t*, the correct temporal alignment of the simulation results is of high importance as well. As we experienced during testing, already a small shift in the alignment of the timing can cause simulations not to converge.

When comparing the novel approach with existing approaches in literature, we can find that partitioned approaches continue to gain in importance. The group around (4) implemented a monolithic solver to tackle the multi physics problem. To account for the influences of the fluid simulation in the mechanics domain, a fluid Cauchy stress tensor is considered. Thus, in each time step, fluid and mechanics equations are solved within one single solver. Like in this work, the NSE are formulated in ALE description. However, when using measurement data as a boundary condition for fluid simulation in the application part, the motion equations are no longer solved. Based on the measured time courses, the deformation of the myocardial walls is known a priori and is not computed based on the monolithic equations any more. This approach still delivered accurate results. Lately, Regazzoni et al. (9) additionally implemented an implicit-explicit scheme based on an inter-grid transfer operator to enable the computation using different mesh resolutions and discretizations in space and time. Their coupling scheme therefore is segregated and staggered. In the fully coupled model suggested by Santiago et al. (34), a partitioned FSI simulation was used. They tackled the multi-physics problem of cardiac simulations by a division in two domains with a Dirichlet-Neumann decomposition each. Based on a strongly coupled staggered approach, the electro-mechanical domain is bidirectionally coupled to the fluid domain. A fluid-structure interface is implemented to compute the discrete position of the nodes based on Degroote et al. (7). Also Viola et al. (10) presented a multi-way coupled computational model based on an immersed boundary grid for the left part of the heart. In their fluid-structure-electrophysiology coupling approach, a central structure evolution equation is solved to link between the three named physical domains. So in each time step, the new mechanical geometry is used as an input for the three single physics solvers. The approach was found to deliver accurate results on an idealized left heart geometry. Another approach suggested by Habchi et al. (35) is also based on a partitioned solver. A block Gauss Seidel implicit scheme is used, but there the three domains fluid, mechanics and mesh motion are considered. Overall, there are a lot of FSI approaches and applications, with most of them based on a partitioned approach. The review of Hirschhorn et al. (5) concisely presents a large number in their corresponding application fields.

Summing up, all approaches listed in this section are based on a staggered scheme. Thus, our approach is based on the same basic decomposition, that is used in established processes. However, as we suggest a cycle to cycle coupling, the effort of implementing an elaborated coupling scheme, that closely couples both physics, is circumvented.

An abstract way to model the influence of the circulatory system is to use a 3D-0D coupled approach (23, 36–38). To adequately define the interface between the circulatory system pressures and the fluid simulation boundary pressures, we introduced the prolonged trunks in the left heart to model the pulmonary veins and the aorta. These rigid tubes also allow for steady state flow conditions.

To replicate realistic blood flow patterns, Daub et al. (28) showed that a comparably simple valve model can be sufficient. Inserting a tripping ring along the valve annulus enhances vortex formation such that the typical vortex ring develops in the left ventricular chamber. In this way, previous limitations of the comparably simple valve model can be overcome (39, 40). In the present study, the valve planes between atria and ventricles incorporate a notable narrowing, which already represents the effective blockage of the valve leaflets considered sufficient to demonstrate the basic performance of the coupling algorithm. The same holds true for the limitation already discussed by Daub et al. (28): This highly efficient numerical representation of the MV may not be sufficient to study MV flow characteristics or MV pathology in detail.

In hemorheology studies, the characteristics of the blood are investigated. It's a matter of debate whether blood can be modeled as a Newtonian fluid, such that the Navier stokes equations as presented above hold true. As for example (41) state, length and time scales are sufficiently large in macroscopic views of the heart chambers, such that the continuum hypothesis holds true. Therefore, we model blood as a Newtonian fluid in this study. However, non-Newtonian effects could potentially play a role in the prolonged trunks (42).

The results presented in this study show that the sequential coupling approach could be successfully applied for a healthy heart model. In particular, the Euclidean distance converged for successive mechanical iterations. A limit cycle was reached after already one iteration and oscillations decreased significantly.

The numerical stability of the novel approach was high for the investigated heart model. However, as mentioned in section 2.4, the pressure factor has an impact on the absolute pressure values and the absolute deviations. Thus, a non-limited scaling factor could lead to instabilities of the mechanical simulation. Especially if the fluid and the mechanical simulation differ strongly, e.g., caused by a time offset, numerical stability can be impaired. The oscillations appearing in the PV loop are probably introduced by the application of the pressure factor: A steady state system that is exposed to a pressure step, in our case the multiplication with a pressure factor, is brought out of balance and starts to oscillate. To cope with this scenario, normally several iterations are computed, until a new steady state is reached. As in our case the circulatory system does not compute several iterations, the discontinuity of the multiplied pressure factor (pressure step) has to be handled. We observed that oscillations decreased in a subsequent coupling iteration. Pressure discontinuities in the fluid simulation that arise locally, i.e., in neighboring elements could lead to instabilities in the subsequent mechanical simulation. These local pressure discontinuities could for example be caused by a wrong labeling of cells: As the pressure factor is computed based on the mean pressure in each chamber, the correct classification of cells is of high importance, especially at the edges of neighboring regions.

With a mean Euclidean distance of 0.06 mm in the LV, the tissue deformation resulting from the fluid pressure field is considerably low. Especially compared to the uncertainty that is introduced during the imaging process, during image segmentation and during mesh creation, the mean deviation is neglectable. With the same reasoning, the resulting volume alteration of below 1 ml can be neglected compared to the overall chamber volumes. Thus, the additional effort of a fully coupled simulation is likely not justified. Nevertheless, it can be argued that the maximum geometrical alteration of up to 2 mm is in the same order of magnitude as the imaging uncertainty. This is true but the maximum deviation is limited to a very small number of nodes in time and space. In the regions around the aorta and the apex, fluid dynamics had a higher influence on the model displacement at characteristic points in time (Figure 7).

We contextualize these deviations with an increased blood flow velocity. As increased blood flow results in a lower static wall pressure, it deflects the wall to a lower extent than areas in a mean flow range. This phenomenon is expected to occur in the area around the aorta during ventricular systole, when the blood is ejected out of the LV. As we observe high blood velocities (up to 200 ml/s) in the aortic region, we would expect the fluid to have an influence on the mechanical deformation through FSI. During the ventricular filling phase, the area around the apex is affected by this phenomenon. As the scalar transport confirms, there is a jet toward the apex. This jet also accounts for a local velocity increase, resulting in a higher Euclidean distance between the nodes.

Thus, if the focus of interest is exactly in the detailed resolution of these regions, the impact of fluid dynamics has to be considered especially at the mentioned characteristic points in time. However, even in this case, it is sufficient to compute one iteration of the sequential pressure-deviation coupling algorithm.

Considering the washout of the LV in Figure 8, the altered mechanical deformation leads to a noticeable increase. This is due to the fact that residual volume is summed up cycle by cycle, which finally leads to an increased washout of 4%. Thus, in the case of simulating subsequent heart cycles, considering one iteration of the sequential coupling procedure would lead to more precise results.

In conclusion, the retrograde influence of fluid dynamics on mechanical deformation appears as not particularly relevant for most of the time of the heart cycle in a healthy configuration. In other cases, one iteration of the fluid-mechanic coupling would be sufficient to account for the minor deviations.

Regarding other coupling approaches, the main advantage of the sequential coupling approach is the independence of the mechanics and fluid solvers. The presented approach enables to use different, completely independent software packages for the simulation of the different physics. The solvers are only linked by exchanging the pressure factor and the only requirement for the mechanical solver is the ability to incorporate the external pressure factor during simulation.

In future work, this iterative coupling approach will have to prove its ability to deliver physiologically accurate results also for diseased heart models. To improve stability of the coupling framework and to increase smoothness of the results, a time window for the determination of the scaling factor *y*_*s*_ can be introduced. Additionally, the degree of automation can be increased toward a fully automatic pipeline.

Summing up, we observed that the influence of fluid dynamic pressure resulted in a structural deviation of up to 2 mm. These deviations could already be resolved after one coupling iteration. In conclusion, the sequential coupling approach with its comparably low computational effort delivered promising results for modeling fluid-structure interaction in cardiac simulations.

## Data Availability Statement

The original contributions presented in the study are included in the article, further inquiries can be directed to the corresponding author.

## Ethics Statement

The studies involving human participants were reviewed and approved by Heidelberg University Hospital (local IRB). The patients/participants provided their written informed consent to participate in this study.

## Author Contributions

AL, OD, BF, LH, TG, EK, and AD designed the study. LH, AD, EK, TG, and JB implemented and tested the pipeline. JB executed the simulations, evaluated the results, and drafted the manuscript. All authors critically revised the manuscript.

## Funding

We acknowledge the financial support from the Federal Ministry of Education and Research of Germany in the framework of the project Towards an integrated numerical heart model (05M16VKA) and the Deutsche Forschungsgemeinschaft (DFG, German Research Foundation, LO 2093/6-1 and Project-ID 258734477–SFB 1173). We furthermore acknowledge support by the KIT-Publication Fund of the Karlsruhe Institute of Technology.

## Conflict of Interest

The authors declare that the research was conducted in the absence of any commercial or financial relationships that could be construed as a potential conflict of interest.

## Publisher's Note

All claims expressed in this article are solely those of the authors and do not necessarily represent those of their affiliated organizations, or those of the publisher, the editors and the reviewers. Any product that may be evaluated in this article, or claim that may be made by its manufacturer, is not guaranteed or endorsed by the publisher.
